# Preface to “Health Promotion in Children and Adolescents through Sport and Physical Activities—2nd Edition”

**DOI:** 10.3390/jfmk6010023

**Published:** 2021-03-01

**Authors:** Antonino Bianco

**Affiliations:** Sport and Exercise Sciences Research Unit, University of Palermo, Via Giovanni Pascoli, 6, 90144 Palermo, Italy; antonino.bianco@unipa.it

The second edition of the Special Issue entitled “Health Promotion in Children and Adolescents through Sport and Physical Activities” has been successfully completed, as expected. As stated in the preface to the first edition, this special issue (SI) was initially intended to address a challenge in this field, but this topic is becoming, over time, an important cornerstone for scientists who are exploring the fascinating subject of pediatric exercise. We open the second edition of this Special Issue with an interesting study protocol described by Ng et al., from the Health Research Institute, University of Limerick. The manuscript presents a useful overview of initiatives designed to encourage people to become more active and increase their awareness of the potential benefits of physical activity (PA). Chulvi-Medrano et al. present a 47-year comparison of lower body muscular power in Spanish boys. The authors conclude that a decline in lower body muscular power occurs in 10–11-year-old Spanish boys, which may be due to the increasing prevalence of sedentary lifestyles across Europe, particularly in the southern regions. Josip Karuc and Marjeta Mišigoj-Duraković investigate the relationship between functional movement (FM) patterns, PA level, and weight status in an average adolescent population. As expected, the authors reinforce the consensus that overweight and obese adolescents exhibit poorer functional movement than normal-weight adolescents. Nemanja Lakicevic, a PhD student from Palermo University, presents evidence of the effects of alcohol consumption on recovery following resistance exercise. The systematic review was publicized on social media and provides an interesting overview of the potentially negative effects of alcohol, particularly in regard to adolescents.

The acute cardiometabolic responses to multi-modal integrative neuromuscular training are reported by a pioneer in the science of modern pediatric exercise. Prof. Avery Faigenbaum is currently regarded as a major contributor in the field, often proposing new ideas and highlighting resistance training as the driving force behind the proper growth and development of coming generations. In line with Faigenbaum et al., Migliano et al. significantly contribute to this SI with their validation of cardiorespiratory fitness measurements in adolescents living in Texas (USA). Mustafa Söğüt et al. report interesting data regarding anthropometric obesity indices, body fat percentage, and grip strength in young adults. Of interest, Roscoe et al. investigate accelerometer-based physical activity levels in British preschoolers. On the other hand, joint mobility among young people participating in free climbing has been investigated by Gasbarro et al.

The ESA Program is also discussed in this Special Issue, in the contribution of Ewan Thomas et al., who consider the entire consortium involved in this innovative and sustainable European project (more than 60 people involved). The randomized controlled trial carried out by Chua et al. shows that four minutes of sprint interval training has no acute positive effects on alertness, mood, and memory in children. The study also provides useful information for physical education teachers interested in introducing new settings and scenarios to their lessons. The closing paper of the Special Issue is that by Tamara Rial and colleagues. Their investigation considers urinary incontinence (UI) among adolescent female athletes. Interestingly, the prevalence of UI among such athletes participating in impact sports was most dominant and higher in those engaged in trampolining, followed by rope skipping. In conclusion, I wish to thank the MDPI Editorial Office, and particularly Molly Lu, for supporting me in the role of SI editor. I am also grateful to the EIC Giuseppe Musumeci for the valuable guidance he provided following several rejections.

This Special Issue presents a total of 12 papers, encompassing 31 different affiliations, with authors from 12 different countries spanning three different regions of the world (Europe, North America, and Asia).

I hope to continue the success of this Special Issue with a third edition in the near future.

